# Synergistic Interaction Between *VyMYB24* from Chinese Wild Grape *Vitis yeshanensis* and Cytokinin Enhances Cold Tolerance in Transgenic Tobacco

**DOI:** 10.3390/plants14243777

**Published:** 2025-12-11

**Authors:** Kaiwei Li, Yihai Lu, Jiaxin Li, Yongmu Li, Ling Wang, Ziguo Zhu, Xiucai Fan, Guirong Li

**Affiliations:** 1 School of Horticulture and Landscape Architecture, Henan Institute of Science and Technology, Xinxiang 453003, China; lkw00lucky@163.com (K.L.); lllyihai@163.com (Y.L.); dazjydb@163.com (J.L.); lymeleslbll@163.com (Y.L.); wangling@hist.edu.cn (L.W.); 2 Henan Province Engineering Research Centers of Horticultural Plant Resource Utilization and Germplasm Enhancement, Xinxiang 453003, China; 3 Shandong Academy of Grape, Shandong Academy of Agricultural Sciences, Jinan 370112, China; shanhong98@163.com; 4 Zhengzhou Fruit Research Institute, Chinese Academy of Agricultural Sciences, Zhengzhou 450009, China

**Keywords:** *VyMYB24*, cytokinin, low temperature stress, synergism, transgenic tobacco

## Abstract

Low temperatures severely restrict plant growth and agricultural productivity, and exploring cold tolerance mechanisms is critical. This study investigated the combined effects of grape-derived transcription factor gene *VyMYB24* and the synthetic cytokinin CPPU on cold tolerance in transgenic tobacco. Under low-temperature stress, tobacco plants overexpressing *VyMYB24* and treated with CPPU exhibited significantly alleviated wilting, higher chlorophyll contents, and an improved net photosynthetic rate compared to controls. These plants also showed a lower relative conductivity and malondialdehyde (MDA) content, higher proline accumulation, and elevated activities of superoxide dismutase (SOD), peroxidase (POD), and catalase (CAT), accompanied by reduced reactive oxygen species (ROS), hydrogen peroxide (H_2_O_2_), and superoxide anions (O_2_$\overset{-}{\cdot }$). The results confirm that *VyMYB24* and CPPU synergistically improve cold tolerance via membrane stabilization, enhanced antioxidant defense, and maintained photosynthetic capacity, providing a theoretical foundation for the rational application of CPPU in the cultivation and management of grapes under low-temperature conditions.

## 1. Introduction

Grape (*Vitis* spp.) is a globally important fruit crop with significant economic value. Currently, the most widely cultivated cultivars with desirable fruit quality are predominantly European grapes (*Vitis vinifera* L.), yet their limited cold tolerance poses a major constraint to the sustainable development of the grape industry. As a primary center of grape origin, China harbors abundant wild grape germplasm resources rich in stress-resistant genes [1]. Among them, *Vitis yeshanensis*, commonly known as ‘Yanshan’, exhibits remarkable cold hardiness: its branches can tolerate temperatures as low as −35 °C, and its roots survive between −14 °C and −16 °C, ranking second only to *Vitis amurensis* Rupr. in cold resistance [2]. In addition to its strong cold tolerance, the ‘Yanshan’ grape produces significantly larger fruits than *V. amurensis*, rendering it highly suitable for cross-breeding and genetic studies aimed at enhancing cold resistance in grape hybrids [3].

Cold stress, typically induced by low temperatures above 0 °C, disrupts normal physiological metabolism, cellular homeostasis, and multiple physiological processes, ultimately leading to aberrant plant growth [4]. Short-term exposure results in wilting and leaf lesions, while prolonged cold stress induces metabolic dysfunction, suppresses growth and development, damages reproductive organs, and reduces seed set, thereby causing substantial yield losses in agricultural production [5]. Plants counteract cold stress primarily through the genetic regulation of metabolic pathways, with transcription factors playing a central role in cold signal transduction [6].

The MYB gene family represents one of the largest families of transcription factors in plants, and it plays critical roles in growth and development, cell morphogenesis, organ differentiation, secondary metabolism, and stress responses [7,8,9]. Agarwal et al. [10] demonstrated that *AtMYB15* interacts with ICE1—a bHLH-type transcription factor and core regulator of cold acclimation—to bind specifically to the MYB cis-element in the promoter of CBF genes, thereby participating in the cold stress response. Jin et al. [11] recently reported a similar function in tomatoes: the overexpression of *SpMYB1* (an R2R3-MYB from Solanum pennellii) enhanced cold tolerance by maintaining chlorophyll content, activating antioxidant enzyme genes (*SlCAT*, *SlSOD*), and upregulating stress-responsive genes (SlDREB2), which is consistent with the conserved role of R2R3-MYB transcription factors in plant cold stress responses [11]. Similarly, Laura et al. [12] reported that *OsMYB4* enhances cold tolerance in rice by directly or indirectly regulating downstream target genes. Wang et al. [13] found that the apple MdMYB108L gene is induced by both light and low temperature, and its overexpression enhances cold tolerance in apple callus, underscoring the key role of R2R3-MYB transcription factors in plant responses to low-temperature stress.

Research on cytokinins (CTKs) spans more than a century. Since their isolation and identification by Skoog and Miller in 1955, cytokinins have been recognized as key signaling molecules widely applied in viticulture to promote root, stem, and leaf growth, fruit development, and fruit set [14]. Beyond their roles in growth and development, cytokinins are also involved in abiotic stress responses [15,16,17,18]. For instance, exogenous cytokinin application improved cold tolerance in tobacco cell cultures [19]. In rice, treatment with N^6^-benzyladenine (6-BA)—a synthetic cytokinin commonly used in stress studies to modulate growth and enhance abiotic stress tolerance via antioxidant activation and chloroplast stabilization—increased the activities of antioxidant enzymes (SOD, POD, CAT), reduced MDA content and lipid peroxidation, and protected chloroplast integrity under low-temperature stress, thereby promoting seedling growth [20]. In maize, 6-BA significantly boosted the activities of SOD, POD, and CAT and lowered MDA content, thereby improving drought resistance in seedlings [21]. Yan et al. [22] revealed that high salinity triggers plant adaptation by promoting the degradation of key cytokinin signaling components, offering new insights into hormone–gene crosstalk in stress resistance. Shen [23] compared a transgenic rice line overexpressing *OsU496A* (OX-43) with wild-type Oryza sativa ‘Nipponbare’ (WT) and found that exogenous 6-BA altered grain filling physiology and the expression of key enzymes, indicating that *OsU496A* mediates cytokinin-regulated grain filling. Bian et al. [24] cloned the cytokinin response regulator gene VlRR5 from the ‘Kyoho’ grape and showed that its expression was upregulated by exogenous CPPU [N-(2-chloro-4-pyridyl)-N′-phenylurea] but suppressed by the cytokinin biosynthesis inhibitor lovastatin, confirming that VlRR5 is responsive to cytokinin and regulates fruit set in grapes.

Recent studies have shown that cytokinins sustain active cell division in the Arabidopsis shoot apical meristem by fine-tuning the nucleocytoplasmic shuttling of the cell cycle transcription factor MYB3R4, highlighting the essential role of MYB proteins (e.g., MYB3R1, MYB3R4) in maintaining stem cell activity [25]. However, the synergistic interplay between grape MYB transcription factors and cytokinins in regulating growth, development, and stress adaptation remains poorly understood.

In this study, we applied exogenous CPPU—a cytokinin-like plant growth regulator known to promote root, stem, and leaf growth [26]—to both wild-type (WT) and *VyMYB24*-overexpressing transgenic tobacco plants. The *VyMYB24* gene (VIT_14s0066g01090) was previously identified from the transcriptome of the ‘Yanshan’ grape as a drought-induced R2R3-MYB gene [27]. We investigated the roles of *VyMYB24* and CPPU under low-temperature stress and analyzed their synergistic effects on cold tolerance. This work lays a theoretical foundation for understanding *VyMYB24*–cytokinin interactions in ‘Yanshan’ grape cold tolerance and for rational CPPU application in grape cultivation under low temperatures. Although this study primarily relies on physiological and biochemical data from a transgenic tobacco model system, this approach provides robust support for the synergistic mechanism: (1) the comprehensive dataset (covering photosynthesis, osmotic adjustment, and antioxidant defense) offers strong correlative evidence for the *VyMYB24*–CPPU interaction; (2) using tobacco as a model is rational for the initial mechanistic exploration, as it allows for efficient genetic manipulation and controlled stress imposition, providing a reliable platform to dissect gene–hormone interactions before validation in the more complex grape system.

## 2. Results

### 2.1. Synergistic Regulation of Transgenic Tobacco Phenotypes by VyMYB24 and Cytokinin

**Figure 1:**
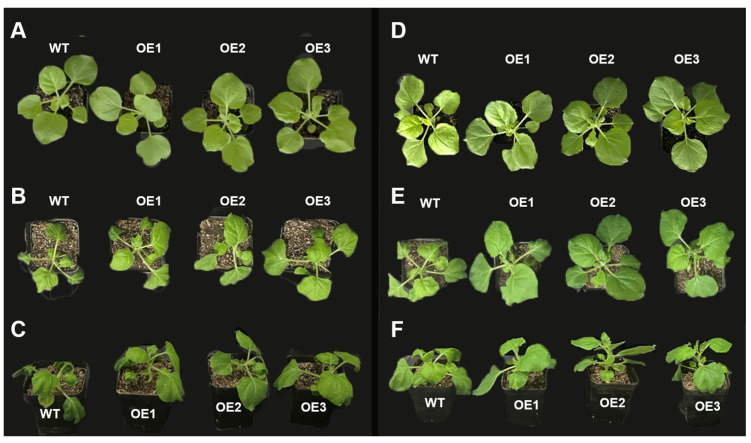
Synergistic enhancement of cold tolerance by *VyMYB24* and cytokinin in transgenic tobacco. (**A**) Top view of water-sprayed controls under normal conditions; (**B**) top and (**C**) front views of water-sprayed plants after 28 h at 0 °C; (**D**) top view of CPPU-sprayed plants under normal conditions, and (**E**) top and (**F**) front views of CPPU-sprayed plants after 28 h at 0 °C. Note: WT, wild-type; OE1/OE2/OE3, independent *VyMYB24*-overexpressing lines.

Cytokinins are known to regulate various aspects of plant growth and development. To evaluate their combined effect with *VyMYB24* under cold stress, we compared the phenotypes of wild-type (WT) and *VyMYB24*-overexpressing (OE) tobacco plants, with or without CPPU treatment. Under normal conditions, both WT and OE plants displayed a similar growth and morphology (Figure 1A,D). After 28 h of low-temperature exposure, severe wilting was observed in WT plants sprayed with water (T_Water/WT_) (Figure 1B,C). In contrast, *VyMYB24*-overexpressing plants without CPPU (T_Water/OE_) exhibited only mild wilting. Notably, OE plants treated with CPPU (T_CPPU/OE_) showed the least phenotypic damage, with leaves maintaining nearly full turgidity (Figure 1E,F).

These results indicate that the combined application of *VyMYB24* overexpression and exogenous CPPU synergistically enhances cold tolerance by maintaining cellular turgor, reducing dehydration, and minimizing growth inhibition—supporting the role of *VyMYB24* and cytokinin in promoting cell division and plant growth under stress.

### 2.2. Synergistic Regulation of Photosynthetic Parameters in Transgenic Tobacco by VyMYB24 and Cytokinin

Low-temperature stress triggered a progressive decline in photosynthetic performance across all tobacco lines; however, this suppression was markedly attenuated in *VyMYB24*-overexpressing plants, especially under CPPU treatment. As shown in Table 1, the total chlorophyll content decreased gradually under prolonged cold exposure, yet the T_CPPU/OE3_ line consistently retained the highest chlorophyll (a + b) levels, reaching 1.601 mg g^−1^ FW by day 28—significantly greater than the 1.121 mg g^−1^ FW in cold-stressed wild-type plants (T_Water/WT_). Notably, even without CPPU, all *VyMYB24*-OE lines maintained a superior chlorophyll retention relative to WT, indicating an intrinsic protective role of *VyMYB24*. Exogenous CPPU further enhanced chlorophyll preservation in OE backgrounds, with T_CPPU/OE2_ and T_CPPU/OE3_ outperforming their water-sprayed OE counterparts across most time points (*p* < 0.05). By contrast, CPPU application in WT plants (T_CPPU/WT_) conferred only limited protection, underscoring that the full chlorophyll-stabilizing effect under cold stress depends on the synergistic action of *VyMYB24* and cytokinin signaling.

A similar synergistic pattern was observed for the net photosynthetic rate (Pn, Table 2). The T_CPPU/OE3_ line exhibited the highest Pn values throughout the stress period, culminating in 8.29 mmol m^−2^ s^−1^ at day 28, significantly exceeding that of T_Water/WT_ (6.23 mmol m^−2^ s^−1^). Both *VyMYB24* overexpression and CPPU contributed to Pn maintenance, with their combination yielding the most robust photosynthetic performance under cold conditions.

Conversely, stomatal conductance (Gs, Table 3) and transpiration rate (Tr, Table 4) were significantly lower in T_CPPU/OE_ plants than in other groups during cold stress. This coordinated reduction in Gs and Tr likely contributed to improved water conservation, mitigating cold-induced dehydration without substantially compromising CO_2_ uptake—a physiological adjustment further reflecting the synergy between *VyMYB24* and cytokinin in optimizing the water–carbon balance under stress.

Collectively, these results demonstrate that *VyMYB24* and CPPU act synergistically to maintain photosynthetic capacity under low-temperature stress, through mechanisms involving chlorophyll protection, enhanced carbon fixation, and optimized stomatal behavior.

### 2.3. Synergistic Regulation of Osmotic Regulators in Transgenic Tobacco by VyMYB24 and Cytokinin

Low-temperature stress significantly disrupted the cellular membrane integrity and osmotic balance; however, these adverse effects were effectively mitigated in the T_CPPU/OE_ plants through the synergistic regulation of key osmotic substances.

**Figure 2:**
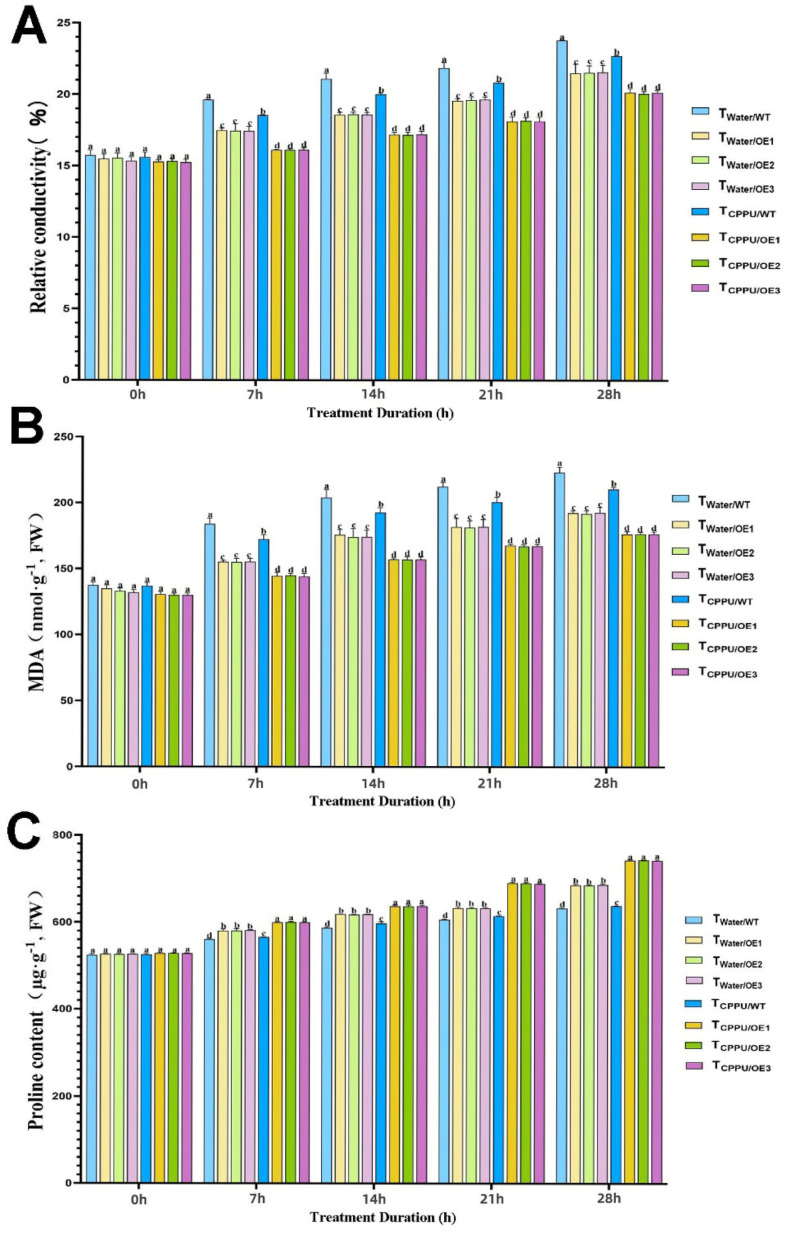
Synergistic regulation of osmotic regulators in transgenic tobacco by *VyMYB24* and cytokinin under low temperatures. (**A**) Relative conductivity; (**B**) MDA content; (**C**) proline content. Data—mean ± SD. Different lowercase letters indicate significant differences (*p* < 0.05). T_Water/WT_: cold-stressed WT, water-sprayed; T_Water/OE_: cold-stressed OE lines, water-sprayed; T_CPPU/WT_: cold-stressed WT, CPPU-sprayed; T_CPPU/OE_: cold-stressed OE lines, CPPU-sprayed.

Cold stress induced a marked increase in the relative electrolyte leakage across all plant groups (Figure 2A). However, the T_CPPU/OE_ line exhibited the least membrane damage, with a significant reduction of approximately 30% in electrolyte leakage compared to the cold-stressed wild-type control T_Water/WT_ by day 28 (*p* < 0.05).

Similarly, the malondialdehyde (MDA) content, an indicator of membrane lipid peroxidation, remained lowest in the T_CPPU/OE_ plants (Figure 2B). At the end of the stress period, the MDA level in this group was about 25% lower than that in T_Water/WT_ plants and was significantly reduced compared to all other treatments, indicating that the combination of *VyMYB24* and CPPU significantly alleviated oxidative membrane damage.

In contrast, proline, an important osmoprotectant, accumulated most substantially in T_CPPU/OE_ plants (Figure 2C). The proline content in this group was approximately 40% higher than in T_Water/WT_ plants during the late stages of stress, and it remained significantly elevated at all time points compared to individual *VyMYB24* overexpression or CPPU application alone. This suggests that the synergistic treatment strongly enhanced the osmotic adjustment capacity.

These results demonstrate that the synergistic action of *VyMYB24* and CPPU effectively minimizes cold-induced membrane damage and optimizes osmotic balance through proline accumulation, which is critical for maintaining cellular homeostasis and improving cold tolerance.

### 2.4. Synergistic Regulation of Antioxidant Enzyme Activities in Transgenic Tobacco by VyMYB24 and Cytokinin

**Figure 3:**
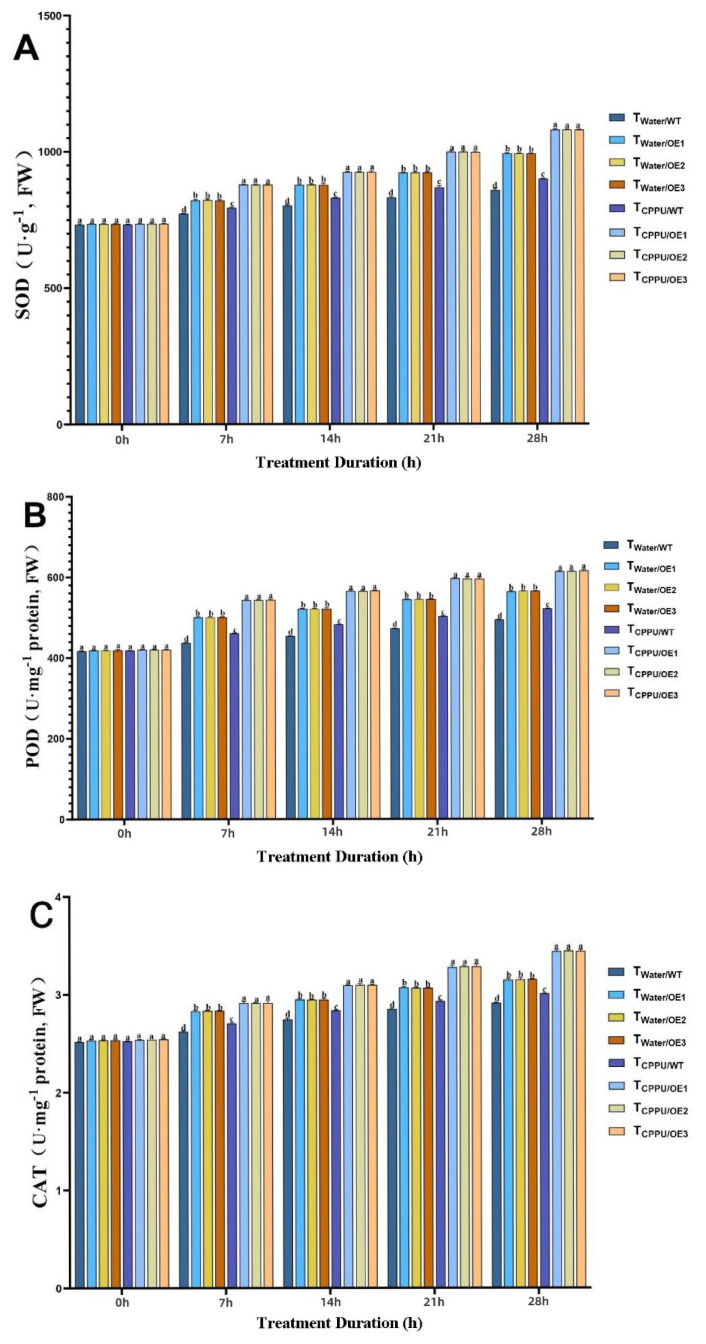
Synergistic regulation of antioxidant enzyme activities in transgenic tobacco by *VyMYB24* and cytokinin under low temperatures. (**A**) SOD; (**B**) POD; (**C**) CAT. Data—mean ± SD. Different lowercase letters indicate significant differences (*p* < 0.05). T_Water/WT_: cold-stressed WT, water-sprayed; T_Water/OE_: cold-stressed OE lines, water-sprayed; T_CPPU/WT_: cold-stressed WT, CPPU-sprayed; T_CPPU/OE_: cold-stressed OE lines, CPPU-sprayed.

Under low-temperature stress, the activities of key antioxidant enzymes—superoxide dismutase (SOD), peroxidase (POD), and catalase (CAT)—were significantly induced in all transgenic tobacco lines. However, the T_CPPU/OE_ plants exhibited the most pronounced enhancement in this enzymatic antioxidant system throughout the treatment period (Figure 3A–C).

SOD activity increased under cold conditions in all groups, but was most markedly elevated in T_CPPU/OE_ plants, reaching a level approximately 35% higher than that in the cold-stressed wild-type control (T_Water/WT_) by the end of the experiment (Figure 3A). A similar trend was observed for POD activity (Figure 3B), which was about 28% greater in T_CPPU/OE_ plants compared to T_Water/WT_, and also significantly surpassed the levels in plants with either *VyMYB24* overexpression or CPPU treatment alone. CAT activity followed this pattern, being roughly 32% higher in the synergistic treatment group than in T_Water/WT_ plants (Figure 3C).

The consistently highest activities of SOD, POD, and CAT in T_CPPU/OE_ plants indicate a robustly potentiated antioxidant capacity, enabling the more efficient scavenging of reactive oxygen species (ROS) and reducing oxidative damage to cellular components under cold stress.

These findings confirm that *VyMYB24* and CPPU act synergistically to activate a coordinated antioxidant enzyme response, which constitutes a critical mechanism in enhancing cold tolerance in transgenic tobacco.

### 2.5. Synergistic Regulation of Oxidative Substances in Transgenic Tobacco by VyMYB24 and Cytokinin

**Figure 4:**
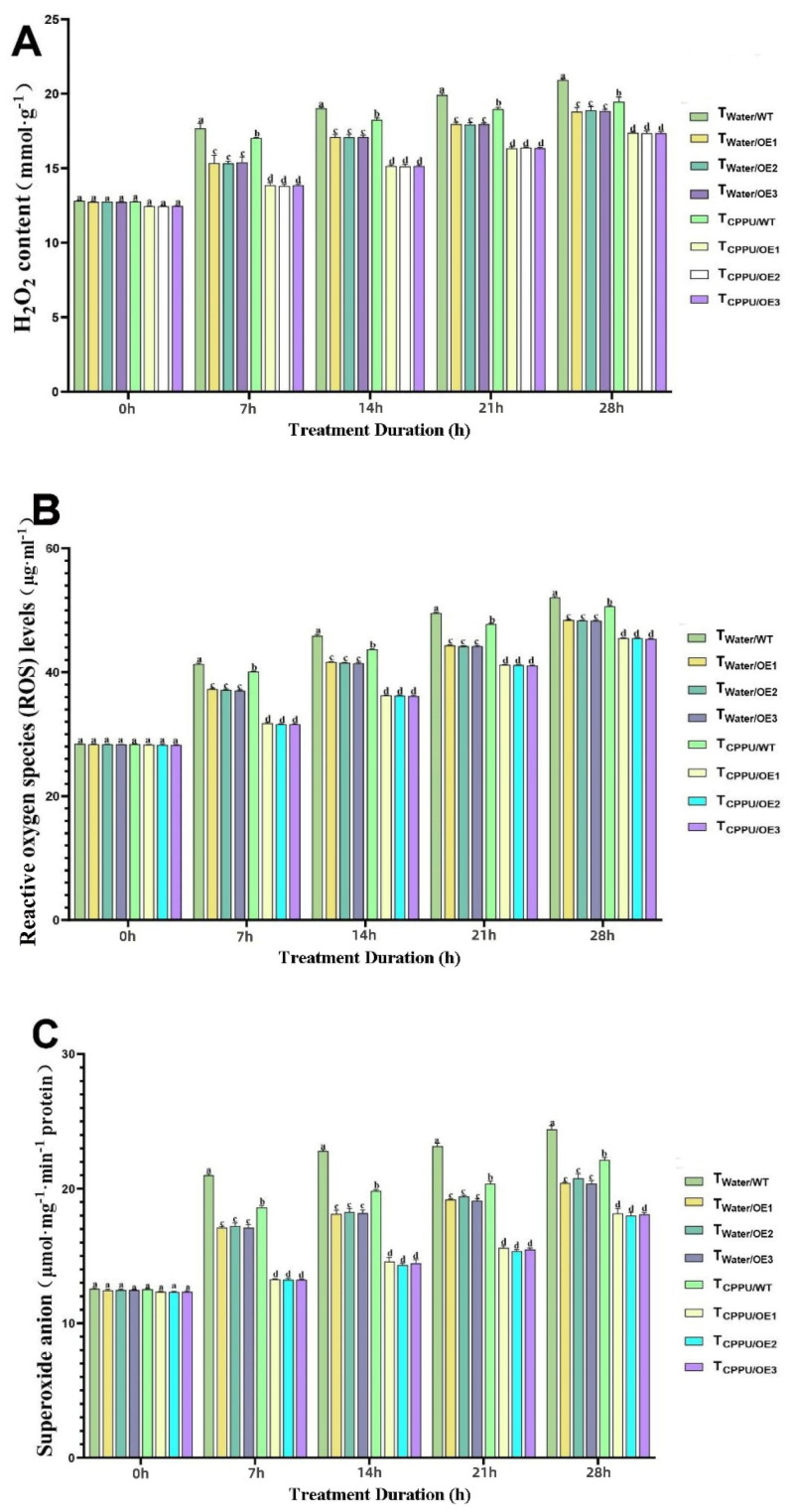
Synergistic regulation of oxidative substances in transgenic tobacco by *VyMYB24* and cytokinin under low temperatures. (**A**) H_2_O_2_ content; (**B**) reactive oxygen species (ROS) levels; (**C**) superoxide anion. Data—mean ± SD (*n* = 3). Different lowercase letters indicate significant differences (*p* < 0.05). T_Water/WT_: cold-stressed WT, water-sprayed; T_Water/OE_: cold-stressed OE lines, water-sprayed; T_CPPU/WT_: cold-stressed WT, CPPU-sprayed; T_CPPU/OE_: cold-stressed OE lines, CPPU-sprayed.

Under cold stress, a significant accumulation of reactive oxygen species (ROS), hydrogen peroxide (H_2_O_2_), and superoxide anions (O_2_$\overset{-}{\cdot }$) was observed in all tobacco plants; however, the T_CPPU/OE_ line displayed the most effective suppression of these oxidative markers (Figure 4A–C).

As shown in Figure 4A, the H_2_O_2_ content remained low under normal conditions but increased progressively during cold exposure. The T_CPPU/OE_ plants consistently maintained the lowest H_2_O_2_ levels, showing a reduction of approximately 40% compared to the cold-stressed wild-type control (T_Water/WT_) at the end of the treatment period. A similar trend was observed for overall ROS levels (Figure 4B), which were about 35% lower in T_CPPU/OE_ plants than in T_Water/WT_, and significantly lower than in all other treatment groups. Furthermore, the rate of O_2_$\overset{-}{\cdot }$ production (Figure 4C) was most effectively restrained in T_CPPU/OE_ plants, being roughly 45% lower than that in T_Water/WT_ plants under sustained low-temperature stress.

These results clearly demonstrate that the synergistic action of *VyMYB24* and CPPU significantly alleviates cold-induced oxidative stress by suppressing the accumulation of H_2_O_2_, ROS, and O_2_$\overset{-}{\cdot }$, thereby contributing to the maintenance of redox homeostasis and cellular integrity under low-temperature conditions.

## 3. Discussion

Plants respond to low-temperature stress through integrated physiological adjustments involving the regulation of photosynthesis, maintenance of osmotic balance, and activation of antioxidant metabolism [28,29]. Notably, similar multi-pathway regulatory patterns mediated by exogenous substances have been reported in grapes: exogenous dopamine (0.4 mmol L^−1^) enhances cold tolerance in the Shine Muscat grape by protecting photosynthetic systems, optimizing ion homeostasis, activating antioxidant enzymes, and upregulating CBF-family cold-responsive genes, further confirming that exogenous regulators can coordinate multiple physiological processes to alleviate low-temperature damage [30]. This study systematically reveals, for the first time, the synergistic effect between the transcription factor gene *VyMYB24* and the exogenous cytokinin CPPU in transgenic tobacco. Echoing the discovery by Agarwal et al. [10] regarding the involvement of *AtMYB15* in the CBF regulatory pathway, our findings demonstrate that *VyMYB24* interacts with cytokinin signaling to establish a multi-level regulatory network. This network synergistically enhances plant cold tolerance by stabilizing photosynthetic performance, improving osmotic adjustment, and strengthening the antioxidant defense system. This transcription factor–hormone synergy offers a novel perspective for understanding the regulatory mechanisms underlying plant responses to low-temperature stress.

Low-temperature stress usually reduces the chlorophyll content by accelerating pig-ment degradation and inhibiting synthesis [31,32]. In our study, T_CPPU/OE_ plants main-tained the highest chlorophyll content and Pn under cold stress, which can be attributed to two synergistic effects: (1) *VyMYB24*-mediated chlorophyll protection: As an R2R3-MYB transcription factor, VyMYB24 may directly bind to the promoters of chlorophyll biosynthesis genes (e.g., CHLH, encoding Mg-chelatase) or repress chlorophyll degradation genes (e.g., SGR, a senescence-associated gene)—consistent with Wang et al. [20], who found MdMYB108L protects chlorophyll under cold stress; (2) CPPU-enhanced chloroplast stability: Cytokinins stabilize chloroplast morphology by upregulating Rubisco activity and protecting thylakoid membrane proteins from cold-induced denaturation [33]. CPPU likely enhances *VyMYB24*’s protective effect by preventing chloroplast swelling, maintaining a light-dependent reaction efficiency. Additionally, T_CPPU/OE_ had lower Gs and Tr, optimizing water use efficiency: it avoids cellular dehydration caused by excessive transpiration while ensuring a sufficient CO_2_ supply for carbon fixation—this ‘water–carbon balance’ aligns with cross-stress tolerance mechanisms in tomatoes under nitrogen deficiency and drought [34].

The relative conductivity, MDA, and proline content reflect plants’ self-protection ability under stress [35,36]. Cold stress induces ROS accumulation (H_2_O_2_, O_2_$\overset{-}{\cdot }$), which damages biofilm structure and function [37,38]. Our results show that T_CPPU/OE_ had the lowest relative conductivity/MDA and highest proline/SOD/POD/CAT activities, confirming a ‘dual protection mechanism’: (1) Membrane stabilization: The lower relative conductivity and MDA indicate reduced membrane leakage and lipid peroxidation—consistent with Gemrotová et al. [39], who found that cytokinins reduce membrane damage under stress. (2) ROS scavenging: The elevated antioxidant enzyme activities directly enhance ROS clearance, while proline acts as a ‘dual-function protectant’ [40]. This mechanism forms a multi-layered defense against cold-induced damage, and is supported by Zong et al. [41], who found that low-concentration 6-BA reduces ROS and MDA content in cold-stressed rice.

Compared to the adaptation mechanism involving cytokinin signaling degradation under salt stress revealed by Yan et al. [22], the *VyMYB24*-CPPU synergy identified in our study presents a distinctly different regulatory mode, suggesting that plants may employ diverse hormonal regulatory strategies to cope with different environmental stresses. Furthermore, referencing the mechanism of the cytokinin-regulated nucleocytoplasmic shuttling of *MYB3R4* reported by Yang et al. [25], we speculate that *VyMYB24* might participate in the low-temperature stress response through similar mechanisms of transcription factor localization control.

Our study focuses on physiological mechanisms of *VyMYB24*-CPPU synergy in tobacco. Although we used tobacco as a model, our results provide a theoretical basis for grape cold resistance research. CPPU is a practical tool for grape cultivation under low temperatures: exogenous CPPU application can alleviate cold damage in grapes—this is particularly valuable in regions prone to spring frosts (e.g., Xinxiang, Henan), where low temperatures cause severe grape yield losses. Future work will (1) verify the mechanism in the ‘Yanshan’ grape (e.g., analyze *VlRR5* and antioxidant enzyme gene expression under *VyMYB24* overexpression and CPPU treatment); (2) identify the direct target genes of *VyMYB24* via ChIP-seq, and explore protein–protein interactions between *VyMYB24* and cytokinin signaling components via co-IP to fully elucidate this regulatory module [10,24,27].

## 4. Materials and Methods

### 4.1. Plant Materials

Wild-type tobacco (*Nicotiana benthamiana* L., WT) and *VyMYB24*-overexpressing transgenic tobacco lines (OE1, OE2, OE3) were obtained from our previous study [27]. All plants were grown in a growth chamber under a 16 h light/8 h dark photoperiod, 25–28 °C, and 70–80% relative humidity (RH).

### 4.2. Low-Temperature Treatment and Exogenous Cytokinin Application

Overexpressed *VyMYB24* transgenic tobacco (OE1, OE2, OE3) plants and wild-type tobacco (WT) plants, with uniform growth 4 weeks after germination, were selected and sprayed with exogenous cytokinin, which comprised 100 mol L^−1^ N-(2-chloro-4-pyridyl)-N′-phenylurea (CPPU) plus Silwet-77 surfactant (final concentration 0.03%, volume ratio). Spraying was performed once daily for 7 consecutive days, with a volume of 10 mL per plant until leaves were uniformly wet without dripping. Controls were sprayed with clean water using the same frequency and volume. And control overexpressed *VyMYB24* transgenic tobacco (OE1, OE2, OE3) plants and WT were sprayed with clean water for 7 days. Low-temperature treatment was administered after 7 days of treatment.

Four treatments were designed. T_CK_ represents the clear water control group, that is, no cytokinin was sprayed; T_CPPU_ represents that cytokinin was sprayed. (1) T_Water/WT_—WT without spraying exogenous cytokinin at low temperatures; (2) T_Water/OE_ (T_Water/OE1_, T_Water/OE2_, T_Water/OE3_)—overexpressed *VyMYB24* transgenic tobacco plants (OE1, OE2, OE3) without exogenous cytokinin sprayed at low temperatures; (3) T_CPPU/WT_—WT sprayed with exogenous cytokinin at low temperatures; (4) T_CPPU/OE_ (T_CPPU/OE1_, T_CPPU/OE2_, T_CPPU/OE3_)—overexpressed *VyMYB24* transgenic tobacco plants (OE1, OE2, OE3) with exogenous cytokinin sprayed at low temperatures. Tobacco leaves were collected at 0 h, 7 h, 14 h, 21 h, and 28 h after 0 °C treatment. Low-temperature treatment was conducted with a BPHJS-120B High and Low Temperature Alternating Humidity Test Kit (Shanghai Yiheng, Shanghai, China) at 0 °C, with a 16/8 h light/dark cycle, light intensity of 2000 lx, and relative humidity of 70–80%. Temperature was monitored every 30 min using built-in sensors to ensure stability (fluctuation ≤ ±0.5 °C). We randomly selected three leaves from the upper-middle part of the plant that were healthy, spot-free, pest-free, and fully mature.

The 0 h sampling time point (before initiating 0 °C treatment) for each group served as its own non-low-temperature control, providing baseline physiological data to quantify the impact of low-temperature stress and the regulatory effects of *VyMYB24* and CPPU. All plants were grown under identical normal conditions (25–28 °C, 16/8 h photoperiod, 70–80% RH) before 0 h sampling, ensuring consistency in initial growth status.

### 4.3. Phenotypic Analysis

Phenotypes of wild-type (WT) and *VyMYB24*-overexpressing transgenic lines (OE1, OE2, OE3) were visually assessed and recorded photographically at 0, 7, 14, 21, and 28 h of low-temperature exposure. The evaluation focused on symptoms of chilling injury, including wilting severity, turgor pressure loss, and the development of chlorosis or necrosis.

### 4.4. Measurement of Photosynthetic Parameters

An LI-6400 portable photosynthetic instrument (Beijing Ligotai Technology Co., Ltd. (Beijing, China)) was used to determine photosynthetic indexes, including the net photosynthetic rate (Pn, mmol m^−2^ s^−1^), stomatal conductance (Gs, mmol m^−2^ s^−1^), and transpiration rate (Tr, g m^−2^ h^−1^). The measuring time was 14:00 on a sunny day; all the parameters for each leaf were measured three times, and the average value was recorded.

### 4.5. Analysis of Physiological and Biochemical Indices

The relative conductivity (RC) was measured using a conductivity meter (DDS-308A, Shanghai Precision Scientific Instrument Co., Ltd. (Shanghai, China)). Relative conductivity = conductivity before boiling water bath R1/conductivity after boiling water bath R2 × 100%. The chlorophyll content, malondialdehyde (MDA) content, proline (Pro) content, superoxide dismutase (SOD) activity, peroxidase (POD) activity, catalase (CAT) activity, hydrogen peroxide (H_2_O_2_) content, and superoxide anion (O_2_$\overset{-}{\cdot }$) production rate (test kit number BC0990, BC0020, BC0290, BC5160, BC0090, BC0200, BC3590, and BC1290, respectively; Beijing Solarbio Technology Co., Ltd. (Beijing, China)) were all determined through spectrophotometry with an ultraviolet–visible spectrophotometer (TU-1810, Beijing Puxi General Instrument Co., Ltd. (Beijing, China)).

### 4.6. Statistical Analysis

All experimental data were derived from three independent biological replicates and are expressed as the mean ± standard deviation (SD). Statistical analyses were performed using SPSS 22.0 (IBM Corporation, Armonk, NY, USA). Significant differences among treatment groups were determined through one-way analysis of variance (ANOVA) followed by Duncan’s multiple range test, with a significance level set at *p* < 0.05. Figures were generated using GraphPad Prism 10.1.2 (GraphPad Software, San Diego, CA, USA).

## 5. Conclusions

Under low-temperature stress, the application of exogenous CPPU reduced the stomatal conductance and transpiration rate in tobacco, thereby minimizing water loss and alleviating wilting symptoms. Concurrently, the combination of *VyMYB24* overexpression and CPPU significantly enhanced the activities of key antioxidant enzymes (SOD, POD, CAT) and promoted proline accumulation, while markedly reducing relative electrolyte leakage, MDA content, and the levels of H_2_O_2_, ROS, and O_2_$\overset{-}{\cdot }$. These coordinated physiological adjustments collectively contributed to an improved cellular membrane stability, mitigated oxidative damage, and supported photosynthetic performance. Notably, the observed improvements were more pronounced in *VyMYB24*-overexpressing transgenic tobacco than in wild-type plants, supporting a synergistic interaction between *VyMYB24* and cytokinin signaling in enhancing low-temperature tolerance. This study provides a theoretical foundation for cold resistance breeding in grapes and supports the rational application of CPPU in grape cultivation under low-temperature conditions.

## Figures and Tables

**Table 1:** Changes in total chlorophyll content (mg g^−1^ FW) of transgenic tobacco under low-temperature treatment.

Treatment	Chlorophyll (a + b) (mg g^−1^ FW)				
	0 d	7 d	14 d	21 d	28 d
T_Water/WT_	1.921 ± 0.157 ^d^	1.721 ± 0.154 ^e^	1.521 ± 0.142 ^f^	1.321 ± 0.142 ^g^	1.121 ± 0.143 ^h^
T_Water/OE1_	2.113 ± 0.153 ^c^	1.713 ± 0.151 ^e^	1.713 ± 0.153 ^e^	1.513 ± 0.143 ^f^	1.313 ± 0.134 ^g^
T_Water/OE2_	2.209 ± 0.157 ^b^	1.809 ± 0.147 ^d^	1.709 ± 0.147 ^d^	1.609 ± 0.144 ^e^	1.409 ± 0.135 ^f^
T_Water/OE3_	2.305 ± 0.152 ^a^	1.905 ± 0.154 ^c^	1.805 ± 0.149 ^c^	1.705 ± 0.147 ^d^	1.505 ± 0.133 ^e^
T_CPPU/WT_	2.075 ± 0.153 ^c^	1.675 ± 0.153 ^e^	1.575 ± 0.151 ^e^	1.475 ± 0.140 ^f^	1.275 ± 0.137 ^g^
T_CPPU/OE1_	2.305 ± 0.154 ^a^	1.905 ± 0.152 ^c^	1.805 ± 0.153 ^c^	1.705 ± 0.141 ^d^	1.505 ± 0.138 ^e^
T_CPPU/OE2_	2.363 ± 0.156 ^a^	1.963 ± 0.148 ^b^	1.863 ± 0.147 ^b^	1.763 ± 0.15 ^c^	1.563 ± 0.136 ^d^
T_CPPU/OE3_	2.401 ± 0.154 ^a^	2.001 ± 0.149 ^a^	1.901 ± 0.146 ^a^	1.801 ± 0.152 ^a^	1.601 ± 0.135 ^b^

Note: Data are means ± SD (*n* = 3). Different lowercase letters indicate significant differences among groups (*p* < 0.05). T_Water/WT_: cold-stressed WT, water-sprayed; T_Water/OE_: cold-stressed OE lines, water-sprayed; T_CPPU/WT_: cold-stressed WT, CPPU-sprayed; T_CPPU/OE_: cold-stressed OE lines, CPPU-sprayed.

**Table 2:** Changes in net photosynthetic rate (Pn, mmol m^−2^ s^−1^) of transgenic tobacco under low-temperature treatment.

Treatment	Net Photosynthetic Rate (Pn) (mmol m^−2^ s^−1^)				
	0 d	7 d	14 d	21 d	28 d
T_water/WT_	8.23 ± 0.85 ^e^	7.73 ± 0.84 ^f^	7.23 ± 0.82 ^g^	6.73 ± 0.72 ^h^	6.23 ± 0.71 ^i^
T_water/OE1_	9.05 ± 0.81 ^d^	8.55 ± 0.83 ^e^	8.05 ± 0.81 ^f^	7.55 ± 0.75 ^g^	7.05 ± 0.65 ^h^
T_water/OE2_	9.46 ± 0.85 ^c^	8.96 ± 0.82 ^d^	8.46 ± 0.83 ^e^	7.96 ± 0.73 ^f^	7.46 ± 0.62 ^g^
T_water/OE3_	9.88 ± 0.86 ^b^	9.38 ± 0.85 ^c^	8.88 ± 0.78 ^d^	8.38 ± 0.75 ^e^	7.88 ± 0.61 ^f^
T_CPPU/WT_	8.89 ± 0.87 ^d^	8.39 ± 0.81 ^e^	7.89 ± 0.79 ^f^	7.39 ± 0.72 ^g^	6.89 ± 0.63 ^h^
T_CPPU/OE1_	9.88 ± 0.83 ^b^	9.38 ± 0.82 ^c^	8.88 ± 0.75 ^d^	8.38 ± 0.74 ^e^	7.88 ± 0.65 ^f^
T_CPPU/OE2_	10.12 ± 0.85 ^a^	9.62 ± 0.83 ^b^	9.12 ± 0.82 ^c^	8.62 ± 0.78 ^d^	8.12 ± 0.64 ^e^
T_CPPU/OE3_	10.29 ± 0.86 ^a^	9.79 ± 0.85 ^a^	9.29 ± 0.75 ^a^	8.79 ± 0.75 ^a^	8.29 ± 0.64 ^b^

Note: Data are means ± SD (*n* = 3). Different lowercase letters indicate significant differences among groups (*p* < 0.05). T_Water/WT_: cold-stressed WT, water-sprayed; T_Water/OE_: cold-stressed OE lines, water-sprayed; T_CPPU/WT_: cold-stressed WT, CPPU-sprayed; T_CPPU/OE_: cold-stressed OE lines, CPPU-sprayed.

**Table 3:** Changes in stomatal conductance (Gs, mmol m^−2^ s^−1^) of transgenic tobacco under low-temperature treatment.

Treatment	Stomatal Conductance (Gs) (mmol m^−2^ s^−1^)				
	0 d	7 d	14 d	21 d	28 d
T_CK/WT_	0.18 ± 0.02 ^c^	0.16 ± 0.02 ^d^	0.14 ± 0.02 ^e^	0.12 ± 0.03 ^f^	0.1 ± 0.03 ^g^
T_CK/OE1_	0.16 ± 0.02 ^d^	0.14 ± 0.02 ^e^	0.12 ± 0.03 ^f^	0.1 ± 0.02 ^g^	0.08 ± 0.02 ^h^
T_CK/OE2_	0.21 ± 0.03 ^a^	0.19 ± 0.03 ^b^	0.17 ± 0.02 ^c^	0.15 ± 0.03 ^d^	0.13 ± 0.03 ^e^
T_CK/OE3_	0.22 ± 0.02 ^a^	0.2 ± 0.02 ^b^	0.18 ± 0.02 ^c^	0.16 ± 0.02 ^d^	0.14 ± 0.02 ^e^
T_CPPU/WT_	0.17 ± 0.03 ^c^	0.15 ± 0.03 ^d^	0.13 ± 0.03 ^e^	0.11 ± 0.03 ^f^	0.09 ± 0.02 ^g^
T_CPPU/OE1_	0.14 ± 0.02 ^e^	0.12 ± 0.02 ^f^	0.1 ± 0.02 ^g^	0.08 ± 0.02 ^h^	0.06 ± 0.03 ^i^
T_CPPU/OE2_	0.13 ± 0.02 ^e^	0.11 ± 0.03 ^f^	0.09 ± 0.03 ^g^	0.07 ± 0.02 ^h^	0.05 ± 0.02 ^i^
T_CPPU/OE3_	0.12 ± 0.02 ^f^	0.1 ± 0.02 ^g^	0.08 ± 0.02 ^h^	0.06 ± 0.02 ^i^	0.04 ± 0.03 ^j^

Note: Data are means ± SD (*n* = 3). Different lowercase letters indicate significant differences among groups (*p* < 0.05). T_Water/WT_: cold-stressed WT, water-sprayed; T_Water/OE_: cold-stressed OE lines, water-sprayed; T_CPPU/WT_: cold-stressed WT, CPPU-sprayed; T_CPPU/OE_: cold-stressed OE lines, CPPU-sprayed.

**Table 4:** Changes in transpiration rate (Tr, g m^−2^ h^−1^) of transgenic tobacco under low-temperature treatment.

Treatment	Transpiration Rate (Tr) (g m^−2^ h^−1^)				
	0 d	7 d	14 d	21 d	28 d
T_CK/WT_	2.24 ± 0.24 ^c^	2.04 ± 0.25 ^d^	1.84 ± 0.24 ^e^	1.64 ± 0.21 ^f^	1.44 ± 0.24 ^g^
T_CK/OE1_	2.02 ± 0.21 ^d^	1.82 ± 0.25 ^e^	1.62 ± 0.21 ^f^	1.42 ± 0.25 ^g^	1.22 ± 0.21 ^h^
T_CK/OE2_	2.58 ± 0.23 ^a^	2.38 ± 0.21 ^b^	2.18 ± 0.23 ^c^	1.98 ± 0.23 ^d^	1.78 ± 0.23 ^e^
T_CK/OE3_	2.69 ± 0.22 ^a^	2.49 ± 0.25 ^a^	2.29 ± 0.23 ^b^	2.09 ± 0.21 ^c^	1.89 ± 0.21 ^d^
T_CPPU/WT_	2.06 ± 0.23 ^d^	1.86 ± 0.21 ^e^	1.66 ± 0.21 ^f^	1.46 ± 0.24 ^g^	1.26 ± 0.24 ^h^
T_CPPU/OE1_	1.79 ± 0.25 ^e^	1.59 ± 0.23 ^f^	1.39 ± 0.25 ^g^	1.19 ± 0.25 ^h^	0.99 ± 0.23 ^i^
T_CPPU/OE2_	1.72 ± 0.21 ^e^	1.52 ± 0.24 ^f^	1.32 ± 0.24 ^g^	1.12 ± 0.21 ^h^	0.92 ± 0.21 ^i^
T_CPPU/OE3_	1.68 ± 0.22 ^e^	1.48 ± 0.21 ^f^	1.28 ± 0.23 ^g^	1.08 ± 0.22 ^h^	0.88 ± 0.25 ^i^

Note: Data are means ± SD (*n* = 3). Different lowercase letters indicate significant differences among groups (*p* < 0.05). T_Water/WT_: cold-stressed WT, water-sprayed; T_Water/OE_: cold-stressed OE lines, water-sprayed; T_CPPU/WT_: cold-stressed WT, CPPU-sprayed; T_CPPU/OE_: cold-stressed OE lines, CPPU-sprayed.

## Data Availability

The original contributions presented in this study are included in the article. Further inquiries can be directed to the corresponding author.

## Abbreviations

6-BA: 6-benzylaminopurine; SOD: superoxide dismutase; POD: peroxidase; CAT: catalase; MDA: malondialdehyde; CPPU: N-(2-chloro-4-Pyridyl)-N′-phenylurea; WT: wild-type tobacco plants; T_Water_: the clear water control group; T_CPPU_: cytokinin was sprayed; T_CK/WT_: WT without spraying exogenous cytokinin at low temperatures; T_Water/OE_: overexpressed *VyMYB24* transgenic tobacco plants; T_CPPU/WT_: WT sprayed with exogenous cytokinin at low temperatures; T_CPPU/OE_: overexpressed *VyMYB24* transgenic tobacco plants (OE1, OE2, OE3) with exogenous cytokinin sprayed at low temperatures.
